# A “Bundle of Care” to Improve Anticoagulation Control in Patients Receiving Warfarin in Uganda and South Africa: Protocol for an Implementation Study

**DOI:** 10.2196/46710

**Published:** 2023-07-19

**Authors:** Andrea L Jorgensen, Catherine Orrell, Catriona Waitt, Cheng-Hock Toh, Christine Sekaggya-Wiltshire, Dyfrig A Hughes, Elizabeth Allen, Emmy Okello, Gayle Tatz, Giovanna Culeddu, Innocent G Asiimwe, Jerome Roy Semakula, Johannes P Mouton, Karen Cohen, Marc Blockman, Mohammed Lamorde, Munir Pirmohamed

**Affiliations:** 1 Department of Health Data Science Institute of Population Health Sciences University of Liverpool Liverpool United Kingdom; 2 Desmond Tutu HIV Centre Institute of Infectious Disease and Molecular Medicine University of Cape Town Cape Town South Africa; 3 Infectious Diseases Institute Makerere University College of Health Sciences Kampala Uganda; 4 Institute of Translational Medicine University of Liverpool Liverpool United Kingdom; 5 Institute of Infection and Global Health University of Liverpool Liverpool United Kingdom; 6 Centre for Health Economics and Medicines Evaluation School of Health Sciences Bangor University Bangor United Kingdom; 7 Division of Clinical Pharmacology Department of Medicine University of Cape Town Cape Town South Africa; 8 Uganda Heart Institute Kampala Uganda; 9 The Wolfson Centre for Personalized Medicine, Department of Pharmacology and Therapeutics Institute of Systems, Molecular and Integrative Biology University of Liverpool Liverpool United Kingdom

**Keywords:** drug monitoring, international normalized ratio, Sub-Saharan Africa, warfarin bundle, warfarin, anticoagulation control

## Abstract

**Background:**

The quality of warfarin anticoagulation among Sub-Saharan African patients is suboptimal. This is due to several factors, including a lack of standardized dosing algorithms, difficulty in providing timely international normalized ratio (INR) results, a lack of patient feedback on their experiences with treatment, a lack of education on adherence, and inadequate knowledge and training of health care workers. Low quality of warfarin anticoagulation, expressed as time in therapeutic range (TTR), is associated with higher adverse event rates, including bleeding and thrombosis, and ultimately, increased morbidity and mortality. Processes and interventions that improve this situation are urgently needed.

**Objective:**

This study aims to evaluate the implementation of the “warfarin bundle,” a package of interventions to improve the quality of anticoagulation and thereby clinical outcomes. The primary outcome for this study is TTR over the initial 3 months of warfarin therapy.

**Methods:**

Patients aged 18 years or older who are newly initiated on warfarin for venous thromboembolism, atrial fibrillation, or valvular heart disease will be enrolled and followed up for 3 months at clinics in Cape Town, South Africa, and Kampala, Uganda, where the warfarin bundle is implemented. A retrospective review of the clinical records of patients on warfarin treatment before implementation (controls) will be used for comparison. This study uses a mixed methods approach of the implementation of patient- and process-centered activities to improve the quality of anticoagulation. Patient-centered activities include the use of clinical dosing algorithms, adherence support, and root cause analysis, whereas process-centered activities include point-of-care INR testing, staff training, and patient education and training. We will assess the impact of these interventions by comparing the TTR and safety outcomes across the 2 groups, as well as the cost-effectiveness and acceptability of the package.

**Results:**

We started recruitment in June 2021 and stopped in August 2022, having recruited 167 participants. We obtained ethics approval from the University of Cape Town Faculty of Health Sciences Human Research Ethics Committee, the Provincial Health Research Committees in South Africa, the Joint Clinical Research Centre Institutional Review Board, Kampala, and the University of Liverpool Research Ethics Committee. As of February 2023, data cleaning and formal analysis are underway. We expect to publish the full results by December 2023.

**Conclusions:**

We anticipate that the “bundle of care,” which includes a clinical algorithm to guide individualized dosing of warfarin, will improve INR control and TTR of patients in Uganda and South Africa. We will use these findings to design a larger, multisite clinical trial across several Sub-Saharan African countries.

**International Registered Report Identifier (IRRID):**

DERR1-10.2196/46710

## Introduction

### Background

Oral anticoagulation is vital for several diseases that are prevalent and increasing in Sub-Saharan Africa (SSA). Warfarin remains one of the most frequently used oral anticoagulants, particularly in lower-resource settings, such as SSA [1], but is difficult to use because of its narrow therapeutic index, wide variability in individual daily dose requirements required to achieve the desired effect and need for frequent monitoring. Achievement of the target international normalized ratio (INR) is critical to prevent the recurrence of thrombosis and bleeding. Several studies have described low time in therapeutic range (TTR) [2]. In our previous study, our own cohorts in Uganda and South Africa demonstrated a TTR of 41% [3] compared to TTRs of up to 70% in high-income settings [4].

We identified several reasons that contributed to this low quality of anticoagulation, including a lack of standardized dosing algorithms with different practices in different clinics, difficulty in providing INR results at the time patients attend clinic, a lack of patient feedback on their experiences while on treatment and education on adherence, inadequate knowledge and training of health care workers, a lack of standardization of quality improvement procedures, and the lack of availability of different dosage strengths of warfarin tablets.

A simulation study [5] in the United States suggested that improving TTR by 5% resulted in a 7.2% reduction in all adverse events in a population of 67,070 patients anticoagulated for atrial fibrillation over a 2-year period. This included 183 ischemic strokes, 269 major hemorrhages, and 662 deaths. Increasing TTR by 5% saved US $15.9 million and gained 863 quality-adjusted life years (QALYs) during this period [5]. In our population, with a median TTR of 41% [3], an improvement in TTR would be expected to have a greater impact since the relationship between TTR and mortality is nonlinear.

We aim to introduce a package of measures using a mixed methods approach, which we have termed the “warfarin bundle,” in anticoagulation clinics in 2 cities (Cape Town, South Africa, and Kampala, Uganda), to improve the quality of anticoagulation. The bundle includes patient-centered and process-centered activities, namely (1) implementing a clinical warfarin dosing algorithm, (2) providing adherence support, (3) conducting root-cause analysis of warfarin-related adverse events, (4) implementing point-of-care INR testing, and (5) providing staff training. We anticipate that this warfarin bundle will be a cost-effective approach for better INR control compared to the current standard of care.

### Hypothesis

Our underlying hypothesis is that implementing a bundle of measures, including a clinical algorithm to guide individualized dosing of warfarin, will improve INR control and TTR in patients in Uganda and South Africa.

### Objectives

The primary objective of this study is to evaluate whether implementation of the warfarin bundle improves TTR. Secondary objectives include evaluating whether implementation of the warfarin bundle improves time to achieving a therapeutic INR or reduces the occurrence of adverse effects, assessing the acceptability of the bundle, exploring patients’ experiences, and estimating cost-effectiveness.

## Methods

### Study Design and Setting

This is a mixed methods implementation study of patient- and process-centered activities to improve the quality of anticoagulation in Sub-Saharan African patients involving 3 hospitals in Kampala, Uganda (Mulago National Referral Hospital, Uganda Heart Institute, and Kiruddu National Referral Hospital) and 2 hospitals in Cape Town, South Africa (Groote Schuur Hospital and Tygerberg Hospital).

**Figure 1:**
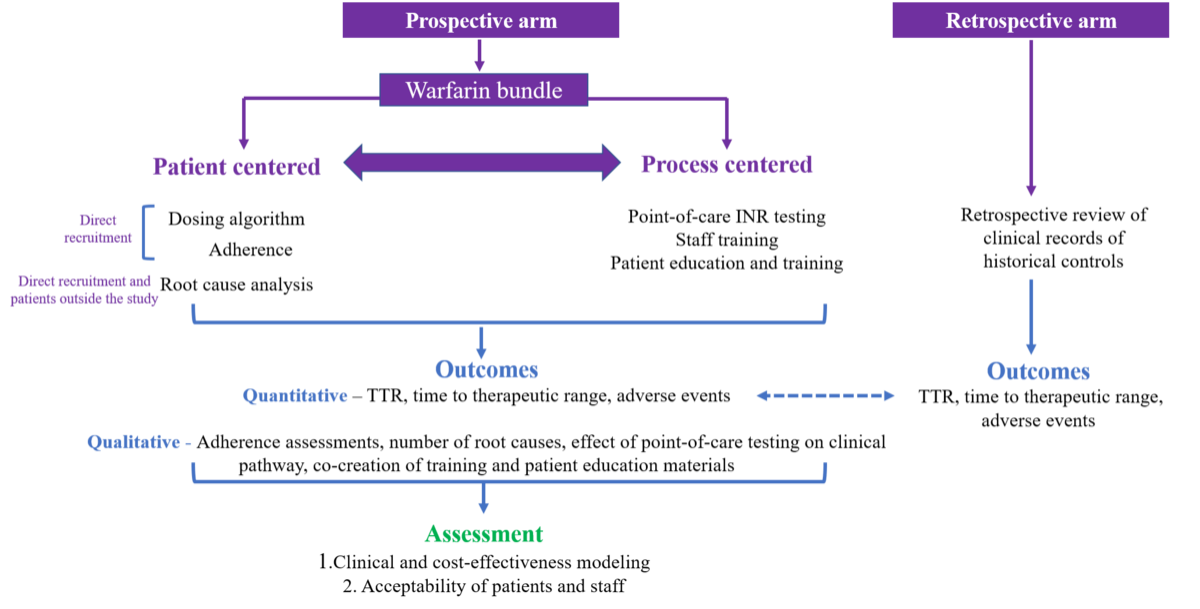
Study design highlighting prospective and retrospective components and outcomes. INR: international normalized ratio; TTR: time in therapeutic range.

The general study design is described in Figure 1.

### Patient Centered Components of the Warfarin Bundle

#### Implementing the Use of Dosing Algorithms

Participants of Black African ancestry will be dosed according to the warfarin dose initiation algorithm developed in our preceding study in Black African patients in Uganda and South Africa, which takes account of patient age, weight, target INR, and HIV status [6] (Multimedia Appendix 1). In South Africa, patients of other ethnicities will be initiated on warfarin using warfarin dosing algorithms which have already been published [7,8] (Multimedia Appendix 2). We will collaborate with their treating clinicians and calculate a warfarin initiation dose using the relevant algorithm.

Day 0 is considered the first day that warfarin is taken. We recruited participant up to 7 days before and no more than 1 day after warfarin was initiated, meaning some individuals may have taken a single warfarin dose. We will use a paper-based dosing table, using dose formulations currently available, which has been developed for implementation (Multimedia Appendix 1).

#### Adherence Support

The package of adherence support offered will include data-driven counseling and interactive weekly text messaging. We will assess adherence by means of a composite of INR values above and below the therapeutic range, TTR, and self-report.

#### Root Cause Analysis

Root cause analysis (RCA) will be undertaken in all patients on warfarin at the INR clinic or hospital who are seen with serious thrombotic or hemorrhagic events. An RCA committee at each clinic, comprising a multidisciplinary team of 4-6 individuals, will review each case using the 9-step process of RCA described by Charles et al [9]. Root causes and corrective actions developed will be categorized on a standardized RCA data collection form to ease analysis [10]. The results of each RCA (root causes, contributing factors, and corrective actions) will be shared with staff at the clinic. Findings of RCA will be discussed at monthly study meetings to inform the overall warfarin bundle implementation (in areas such as adherence, monitoring, and education).

### Process-Centered Components of Warfarin Bundle

#### Point-of-Care Testing

We will make point-of-care INR testing a central part of improving the clinical pathway. Currently, some patients must wait at least a day to receive their INR result and subsequent dosing guidance.

#### Staff Training

We will include co-design workshops discussing the rationale for the bundle and likely implementation issues with a team of clinicians involved in the provision of anticoagulation services. Training will be personalized based on local requirements, depending on the site and staff involved.

#### Cocreation of Patient Education and Training

Co-design workshops for the development of training material to be used in patient education will bring together patient or community representatives, health care staff, the research team, and a representative from policy makers. Different methods will be discussed and explored by the cocreation team in collaboration with representatives from the community and health care workers to ensure they are applicable to the local setting.

### Participant Eligibility Criteria

Participants will be included in the study if they are adults aged 18 years or older; newly initiated on warfarin anticoagulation for the first time for venous thromboembolism, atrial fibrillation, or valvular heart disease; provide informed consent; and are willing to comply with study procedures. We will exclude participants who are on treatment for tuberculosis, pregnant women, and patients who have had more than one dose of warfarin. For the retrospective study, we will include adults newly initiated on warfarin for more than 3 months before we implement the trial.

### Sample Size and Power Calculations

To calculate our sample size, we assumed TTR in current practice to be 0.41 (based on average TTR from our audit) and the SD of TTR to be 0.25 on the basis that anticoagulation control before implementing the bundle is better than that found during the audit (0.33), due to the introduction of the clinical algorithm in a preceding pilot implementation study. We also assumed a Type I error rate of 0.05, power of 80%, and that the retrospective and prospective ratio would be 2:1. To achieve a minimum clinically important difference of 0.07 (ie, improvement from 43% to 50% TTR), we calculated that we would need at least 304 retrospective and 152 prospective participants (including a 10% contingency for losses to follow-up). This is in total across both Uganda and South Africa.

### Participant Recruitment

#### Prospective Study

**Figure 2:**
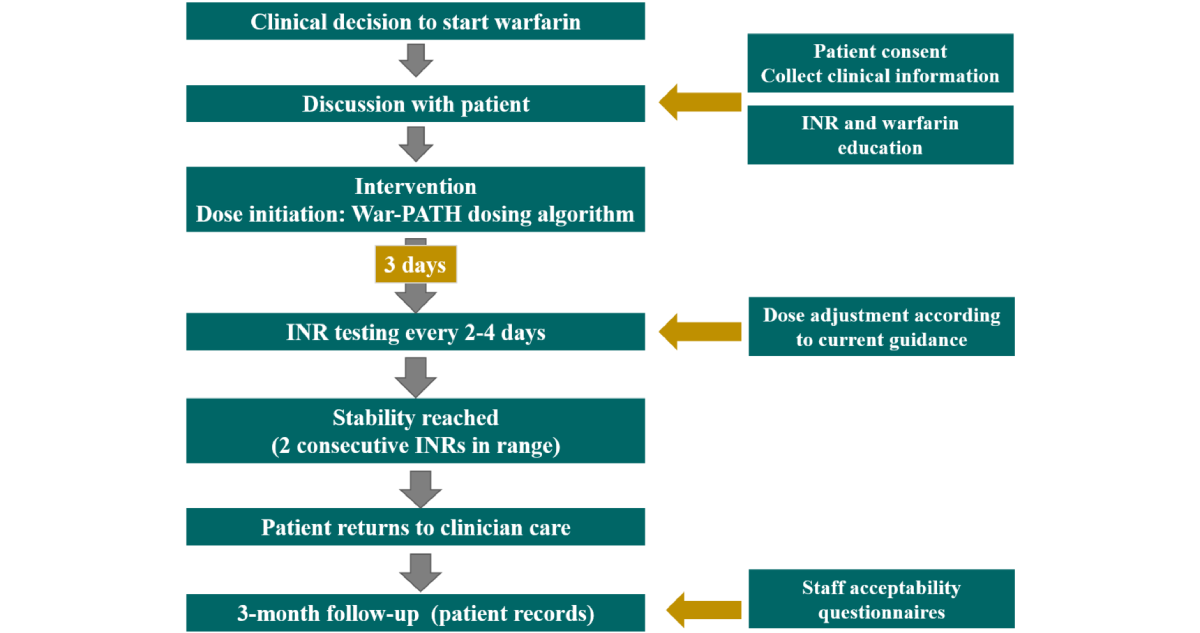
Flow diagram of prospective recruitment and follow-up. INR: international normalized ratio. War-PATH: Warfarin Anticoagulation in Patients in Sub-Saharan Africa.

We will recruit participants for whom a decision has already been made to initiate warfarin by the managing clinician (Figure 2). Decisions regarding whether or not patients require inpatient management for the initiation of warfarin are also made by the managing clinician. Participants who meet the inclusion criteria will be consecutively screened and enrolled in the study. In cases where participants are discharged from the hospital and referred for follow-up in a clinic close to their home or in the outpatient clinic of the hospital, the clinical records used at the recruitment site and those at the referral clinic will be reviewed.

The participant’s first 3 days of dosing will be driven by our new algorithm (Multimedia Appendix 1). Thereafter, dose revisions will be made according to current guidance outlined in the “Standard Treatment Guidelines and Essential Medicines List for South Africa” [11]. We will repeat the INR of participants every 2-4 days until stability is reached (2 consecutive INRs are in the target range). We will ensure close monitoring during the first 14 days of treatment. If stability is reached before the 14th day, we will continue to closely monitor the patient for the first 14 days. If the participant has not reached stability at 14 days, we will continue to monitor the participant with INRs every 2-4 days and adjust the warfarin dose until stability is reached. As we measure participants’ INR every 2-4 days over this period of close follow-up, we will propose dose adjustments to the clinical team based on INR results. The clinical team will still be free to override proposed adjustments based on their clinical expertise. Once stability is reached, we will perform one more INR 1 week later, after which the participants’ care will be fully handed back to the clinical team, and participants will continue to follow-up according to the local standard of care. We will continue to monitor and record all consecutive INR results for 3 months through direct contact with the participant at outpatient clinic, home visits, or electronic or paper record review.

We will collect the following data: age, sex, weight, height, indication for anticoagulation, target INR range, HIV status, recruitment center, all INR measurements during the first 3 months and the first INR after completion of the first 3 months, any dose changes, and any hospitalizations or adverse events (Table 1). We will also complete the EQ-5D-3L questionnaire and a patient-cost questionnaire to enable us to measure changes in quality of life and out-of-pocket expenditures, respectively.

We will test all patients for genetic variants that are determinants of warfarin dose and elucidate whether any patients who were poorly controlled on warfarin had a genetic profile which may have adversely affected their INR control. The genetic testing will be done at the end of the study and will also help with our future plans to validate a genetic dosing algorithm derived from our previous study’s analysis of genome-wide data. (South Africa HREC: 672/2017, Uganda: JC3017). We will also obtain consent to undertake genome-wide analysis in this patient group, which will help in future meta-analyses with our previous studies’ data to identify other genetic loci.

**Table 1:** Participant groups.

	Prospective	Retrospective
Population	Participants starting warfarin and consenting to implementation bundle	Historical controls started on warfarin at anticoagulation clinics where warfarin bundle is implemented
Sample size	152	330
Inclusion criteria	New initiators on warfarin Indication for warfarin is VTE^a^, AF^b^, or VHD^c^ Adults (ie, 18 years or older) Participant is resident in and willing to be followed up at a health facility where follow-up is feasible	New initiators on warfarin Indication for warfarin is VTE, AF, or VHD Adults (ie, 18 years or older)
Exclusion criteria	Pregnancy Received more than one dose of warfarin prior to recruitment Diagnosed with and currently on treatment for Tuberculosis	Pregnancy Diagnosed with and treated for Tuberculosis at time of warfarin initiation
Self-reported ethnicity	Yes	No
EQ-5D-3L questionnaire	Yes	No
Participant expenses tool	Yes	No
Baseline INR^d^	Yes	Yes
INRs during the first 3 months of treatment	Yes	Yes
All adverse events	Yes	Yes
Hospital admissions	Yes	Yes

^a^VTE: venous thromboembolism.

^b^AF: atrial fibrillation.

^c^VHD: valvular heart disease.

^d^INR: international normalized ratio.

#### Retrospective Review of Patients’ Clinical Records

The following data will be collected from clinical records: age, sex, weight, indication for anticoagulation, target INR range, HIV status, recruitment center, all INR measurements during the first 3 months, any dose changes, and any hospitalizations or adverse events. If participants in the retrospective review were initially hospitalized and then discharged, the recruitment hospital site clinical records as well as the clinical records at the referral clinic will be reviewed.

### Withdrawal Criteria

A participant can withdraw from the study at any time if it is his or her wish or if it is medically necessary, as judged by the Investigator. A participant will be withdrawn if any of the following applies:

1. Voluntary discontinuation by the participant, who is at any time free to discontinue their participation in the study without prejudice to further treatment.
2. Incorrect enrollment of the participant (ie, the participant does not meet the required inclusion and exclusion criteria).
3. Participant fails to comply with the protocol requirements or fails to cooperate with the investigator.
4. Participants experiencing severe adverse events who return to standard care.

### Sample Collection

Two tubes of blood (10 or 12 ml total) will be collected in ethylenediaminetetraacetic acid (EDTA) tubes and used for DNA extraction. The EDTA blood sample will be stored at either –20 C or –80 C. One tube will be shipped to the United Kingdom, and 1 kept in Cape Town or Kampala. DNA will be extracted from the EDTA sample at the Wolfson Centre for Personalised Medicine in Liverpool. We will seek ethical approval from the University of Cape Town HREC/Joint Clinical Research Centre Institutional Review Board Office, Kampala, for any future use.

### Adverse Events

The decision to start warfarin will already have been made by participants’ clinical teams before enrollment. We will capture warfarin-related adverse events as part of the secondary outcome measures. A blood test to obtain DNA may sometimes lead to bruising at the site of venepuncture, but all blood will be taken by trained and experienced staff. All adverse events will be recorded and appropriately reported. Investigators will assess whether adverse events were related to warfarin using the Liverpool Adverse Drug Reaction Causality Assessment Tool [12].

### Cost-Effectiveness

We will perform a cost-effectiveness analysis to judge whether the warfarin bundle represents good value for money. These will be based on estimated costs associated with warfarin treatment and health outcomes expressed as QALYs and disability-adjusted life years (DALYs). Resource use will include hospital admissions, outpatient clinic visits, phlebotomy, Point-of-Care and laboratory INR tests, additional blood tests, and prescribed warfarin. External data (which may require extrapolation from other studies or countries) may be required to estimate the costs of managing rare adverse events, including strokes and major bleeds. Unit costs will be identified from a number of sources, including the Western Cape Department of Health (eg, Uniform Patient Fee Schedule), the Ugandan Joint Medical Stores catalog, and the World Health Organization “Choosing Interventions that are Cost-Effective” (WHO-CHOICE) model for estimating unit costs for hospitalizations [13]. Patients will be asked to complete the EQ-5D-3L to estimate health utilities at the initial visit and 1 follow-up visit and to complete a questionnaire to record out-of-pocket costs by telephone. The economic analysis will estimate the incremental costs per QALY gained and DALY averted and inform policy.

### Study Outcomes

The primary outcome for this study is TTR over the initial 3 months of warfarin therapy, which we will measure using the Rosendaal linear interpolation method [14]. We will compare time in the therapeutic INR range between the prospectively followed participants who receive the warfarin bundle and a retrospectively reviewed patient group. This will yield an estimate of the impact of the warfarin care bundle as a whole. It will not be possible to distinguish the effect of the individual components of the warfarin bundle on TTR.

The secondary outcomes include time to achieve therapeutic INR and number of adverse events (death, bleeding, or thrombosis), which will be compared between the prospectively followed-up participants and the retrospectively reviewed patient group. We will also explore staff and patient experiences and acceptability of the warfarin bundle using in-depth interviews or focus group discussions and conduct an economic analysis of the cost-effectiveness of the warfarin bundle. In addition, we will perform a qualitative assessment of useful lessons from the RCA.

### Patient and Public Involvement

Patients or the public will not be involved in the design, conduct, or reporting of this study. However, where possible, study findings will be disseminated through public and patient engagement activities.

### Data Analysis Plan

For the prospective and retrospective studies, all statistical analyses will be undertaken in R. For determining percentage time in target range for each participant, all INR measurements during the first 12 weeks as well as the first INR measurement after the 12-week time point will be used, and the method of Rosendaal et al [14] will be applied. Percentage TTR will be compared between the prospective implementation group and the retrospective standard of care group using the Student *t* test. Time to achieving target INR (defined as the first of at least two INR measurements in range at the same dose) will be compared between the prospective and retrospective groups using the log-rank test (to compare the survival distributions of the 2 groups, and these will be calculated using the Kaplan-Meier estimator). Adverse events will be compared between the 2 groups using a chi-square test. A significance threshold of *P*<.05 will be applied.

The primary analysis will include all participants who remain in the project for at least 2 weeks (14 days). Our primary analysis will include only patients who have at least two weeks’ follow-up, since it is unlikely that those with less than 2 weeks’ follow-up will have sufficient information on INR measurements for TTR to be reliably estimated. A sensitivity analysis will be undertaken, including only participants who have completed 12 weeks (84 days) of follow-up.

We will also conduct both an intention-to-treat analysis (including all recruited patients) and a per-protocol analysis (excluding patients where health care provider decided to reject the dose recommended by the dosing algorithm). The project will follow national guidelines for best practice and be guided by local policy. Relevant data will be made openly available to other researchers in a timely way (after primary outputs are published) with as few restrictions as possible unless we are protecting intellectual property, respecting confidentiality, or honoring third-party agreements. The program management board will review access to data and will provide a governance mechanism.

We will perform a secondary analysis comparing the primary and secondary outcomes between retrospective controls from the pre–COVID-19 and COVID-19 periods to measure the impact of the pandemic on anticoagulation control.

Total direct costs will be calculated for each patient over the course of the 12 weeks. Utilities will be estimated from the administration of the EQ-5D-3L questionnaire (available in 8 languages spoken in South Africa and 2 in Uganda) and valued using the Zimbabwean value set, as none exist for these countries [15]. Regression analyses using transformed utility (or cost) as the dependent variable and controlling for clinical and demographic factors will be used to estimate the association between health utility (or cost) and TTR. These will be incorporated in a decision analytic model, also populated with data from evidence on the relationship between TTR and health outcomes (strokes, hemorrhagic events, etc), to estimate overall costs and QALYs.

In accordance with World Health Organization recommendations, the number of DALYs, expressed as the collective number of years lost due to ill health, disability, or early death, will be calculated by adding the adjusted number of years lived with disability and the number of years of life lost due to premature mortality. The years lived with disability component represents a duration that is scaled by disability severity weights that range from 0 (no adverse impact on quality of life) to 1 (burden equivalent in preference to being dead). Weightings will be applied to time with disability based on disease areas [16] and DALYs for individuals will be calculated and used as an alternative metric of cost-effectiveness (cost per DALY averted).

### Ethical Considerations

The study will be conducted in accordance with the recommendations for physicians involved in research on human subjects adopted by the 18th World Medical Assembly, Helsinki, 1964, and revisions up to and including Fortaleza [17]. We obtained approval from the University of Liverpool Research Ethics Committee (reference number: 9963), the University of Cape Town Faculty of Health Sciences Human Research Ethics Committee (reference number: 710/2020), the Provincial Health Research Committees in South Africa, and the Joint Clinical Research Centre Institutional Review Board, Kampala (reference number: JC2120), before the study commenced.

Each participant in the prospective arm was required to provide written informed consent to enter the study only after a full explanation had been given and an information leaflet had been offered. Signed participant consent was obtained. For participants that were unable to read and write, an impartial witness was present during the informed consent process to witness thumbprint consent. The right of the participant to refuse to participate without giving reasons was respected. All participants were free to withdraw at any time. We did not seek patient consent for the retrospective record review; a waiver of consent was sought from the corresponding ethics committees.

All study data will be deidentified. All documents and logs containing identifiable private information about participants will be kept securely and only accessible by study staff and investigators.

## Results

We started recruitment in June 2021 and stopped in August 2022, having recruited 167 participants. As of February 2023, data cleaning and formal analysis are underway. We expect to publish the full results by December 2023.

## Discussion

### Overview

This protocol will evaluate the implementation of the “warfarin bundle,” a group of interventions aiming to improve the quality of warfarin anticoagulation in SSA. Given the complex and multifaceted challenges contributing to suboptimal anticoagulation in these populations, a mixed methods protocol, including both patient-centered and process-centered activities, was designed.

The study design has several limitations, primarily relating to the complexity of the population, the challenges of investigating anticoagulation in this particular setting, and the selection of a bundle of interventions, including patient and provider education, rather than a single intervention.

In Uganda, anticoagulation services are centralized in Kampala, and there are no control clinics that could be run concurrently in a cluster randomized trial design; within South Africa, selection of appropriate control clinics would necessitate involving another city in addition to Cape Town, thereby bringing unfeasible logistic challenges and cost. Therefore, a before-and-after design was considered most appropriate. We have shown in the preceding Audit study that we can get high-quality data from the case notes [3].

An eligibility criterion for participants in the prospective evaluation is their willingness and ability to comply with study procedures. In practical terms, this excludes individuals who travel long distances from peripheral regions of Uganda to the tertiary facilities where anticoagulation is accessed; this may mean that the participants in the retrospective and prospective arms are not perfectly matched in terms of socioeconomic status. However, the recruitment sites are in the area of highest population density, and the majority of individuals in both the retrospective and prospective arms are anticipated to reside within Kampala and the surrounding Wakiso district.

Through the selection of a bundle of interventions, it will not be possible to attribute improvement to a single element of this. However, this is a complex patient group requiring holistic care, and several important areas have been identified for improvement. The health, economic, and qualitative aspects of the protocol are designed to evaluate the benefits of the bundle as a whole.

### Conclusions

We anticipate that the “bundle of care,” which includes a clinical algorithm to guide individualized dosing of warfarin, will improve INR control and TTR in patients in Uganda and South Africa. This has the potential to reduce the substantial morbidity and mortality seen among individuals requiring oral anticoagulation in these settings. We will use these findings to design a larger, multisite clinical trial in other Sub-Saharan African countries.

## Appendix

Multimedia Appendix 1 Warfarin clinical dosing algorithm for participants of Black African ancestry.

Multimedia Appendix 2 Warfarin dosing algorithms for other ethnicities.

## Abbreviations

DALY: disability-adjusted life year
EDTA: ethylenediaminetetraacetic acid
INR: international normalized ratio
QALY: quality-adjusted life year
RCA: root cause analysis
SSA: Sub-Saharan Africa
TTR: time in therapeutic range

## Data Availability

Relevant data will be made openly available to other researchers in a timely way (after primary outputs are published), with as few restrictions as possible unless we are protecting intellectual property, respecting confidentiality, or honoring third-party agreements. Data sharing is not applicable to this article as no data sets have yet been generated or analyzed.
