# Supplementary material for: A “Bundle of Care” to Improve Anticoagulation Control in Patients Receiving Warfarin in Uganda and South Africa: Protocol for an Implementation Study
Source: JMIR Res Protoc. 2023 Jul 19;12:e46710. doi: 10.2196/46710 (PMC10398551; doi:10.2196/46710)
Supplement: Multimedia Appendix 1 [file resprot_v12i1e46710_app1.docx]

## APPENDIX 1. Warfarin clinical dosing algorithm for participants of Black African ancestry

### Calculating the weekly dose

The equation of the final model is: *Weekly dose in mg = 20.2832 – 0.0656 x Age (years) + 0.2178 x Weight (kg) + 7.3190 (if Target INR range is 2.5 to 3.5)*) *+ 8.7973 (if HIV positive) + 3.4054 (if HIV unknown).*

Doses computed by this equation are then rounded off to the nearest 2.5 mg (half of a 5 mg tablet). We rounded off the predicted dose to the nearest 2.5 mg because 1 mg warfarin tablets are not available in South Africa and have limited availability in Uganda. This gives the rounded weekly dose. However, we need to know how this translates to doses to be given on the first 3 days, which is done as described below.

To calculate the total dose to be given over the first 3 days (in mg, to the nearest 2.5 mg tablet):

- Multiply the rounded weekly dose by 3/7 i.e. 3-day dose = weekly dose (mg) * 3 days/7 days
- Round up to the nearest 2.5 mg tablet (we round up to give the higher doses during the start of the dosing week)

How that total 3-day dose is split over the first 3 days will depend on whether the patient has already received a 5mg dose on day 1 or not. If not, they follow dosing schedule A (below); if they have received the initial 5mg dose, they follow dosing schedule B (below).

#### Dosing schedule A

From the total 3-day dose, allocate 2.5 mg to day 1, then to day 2 and day 3 and back to day 1 and so on until the total 3-day dose is all allocated e.g. if we have 10 mg as the 3-day dose, we will allocate:

- 2.5 mg to day 1 (balance of 7.5 mg)
- 2.5 mg to day 2 (balance of 5 mg)
- 2.5 mg to day 3 (balance of 2.5 mg)
- 2.5 mg to day 1 again (balance of 0 mg – allocation stops)

#### Dosing schedule B

5 mg is already given on day 1 so we can only dose on days 2 and 3. From the total 3-day dose, subtract 5mg to have the 2-day dose. From the total 2-day dose allocate 2.5 mg to day 2, then to day 3 and back to day 2 and so on until the total 2-day dose is all allocated. E.g. if we have 12.5 mg as the 3-day dose and a patient has already been dosed 5 mg on day 1, 2-day dose (day 2 and day 3) = 12.5 mg – 5 mg = 7.5 mg. We will allocate:

- 2.5 mg to day 2 (balance of 5 mg)
- 2.5 mg to day 3 (balance of 2.5 mg)
- 2.5 mg to day 2 again (balance of 0 mg – allocation stops)

#### Dosing schedule

In summary, the dosing equation above (or the paper-based charts below) can be used in combination with the table below to determine the initial 3-day dosing schedule for a patient:

| **Weekly dose (mg)*** | **3-day dose (mg)** | **Schedule A** | **Schedule B (received 5mg on day 0)** |
| --- | --- | --- | --- |
| 27.5 | 12.5 | 5 + 5 + 2.5 | 5 + 5 + 2.5 |
| 30-35 | 15 | 5 + 5 + 5 | 5 + 5 + 5 |
| 37.5-40 | 17.5 | 7.5 + 5 + 5 | 5 + 7.5 + 5 |
| 42.5-45 | 20 | 7.5 + 7.5 + 5 | 5 + 7.5 + 7.5 |
| 47.5-52.5 | 22.5 | 7.5 + 7.5 + 7.5 | 5 + 10 + 7.5 |
| 55-57.5 | 25 | 10 + 7.5 + 7.5 | 5 + 10 + 10 |
| 60-62.5 | 27.5 | 10 + 10 + 7.5 | 5 + 12.5 + 10 |

*Range of predicted doses is 27.5 – 62.5 mg/wk (see paper-based charts below).

### Paper-based charts

These have been constructed based on the age (18 to 87 years) and weight (35 to 150 kg) seen in the 364 Ugandan and South African patients in the development cohort. Patient’s ages and weights need to be rounded off to the nearest multiple of five before these charts can be used (this rounding off can cause a slight discrepancy for some doses – as compared to those calculated by the equation).

#### Target INR range 2.0 to 3.0

HIV_negative

20yrs 25yrs 30yrs 35yrs 40yrs 45yrs 50yrs 55yrs 60yrs 65yrs 70yrs 75yrs 80yrs 85yrs 90yrs

35 kg 27.5 27.5 25.0 25.0 25.0 25.0 25.0 25.0 25.0 22.5 22.5 22.5 22.5 22.5 22.5

40 kg 27.5 27.5 27.5 27.5 27.5 25.0 25.0 25.0 25.0 25.0 25.0 25.0 22.5 22.5 22.5

45 kg 30.0 27.5 27.5 27.5 27.5 27.5 27.5 27.5 25.0 25.0 25.0 25.0 25.0 25.0 25.0

50 kg 30.0 30.0 30.0 30.0 27.5 27.5 27.5 27.5 27.5 27.5 27.5 27.5 25.0 25.0 25.0

55 kg 30.0 30.0 30.0 30.0 30.0 30.0 30.0 27.5 27.5 27.5 27.5 27.5 27.5 27.5 27.5

60 kg 32.5 32.5 32.5 30.0 30.0 30.0 30.0 30.0 30.0 30.0 30.0 27.5 27.5 27.5 27.5

65 kg 32.5 32.5 32.5 32.5 32.5 32.5 30.0 30.0 30.0 30.0 30.0 30.0 30.0 30.0 27.5

70 kg 35.0 35.0 32.5 32.5 32.5 32.5 32.5 32.5 32.5 32.5 30.0 30.0 30.0 30.0 30.0

75 kg 35.0 35.0 35.0 35.0 35.0 32.5 32.5 32.5 32.5 32.5 32.5 32.5 32.5 30.0 30.0

80 kg 37.5 35.0 35.0 35.0 35.0 35.0 35.0 35.0 35.0 32.5 32.5 32.5 32.5 32.5 32.5

85 kg 37.5 37.5 37.5 37.5 35.0 35.0 35.0 35.0 35.0 35.0 35.0 35.0 32.5 32.5 32.5

90 kg 37.5 37.5 37.5 37.5 37.5 37.5 37.5 37.5 35.0 35.0 35.0 35.0 35.0 35.0 35.0

95 kg 40.0 40.0 40.0 37.5 37.5 37.5 37.5 37.5 37.5 37.5 37.5 35.0 35.0 35.0 35.0

100 kg 40.0 40.0 40.0 40.0 40.0 40.0 40.0 37.5 37.5 37.5 37.5 37.5 37.5 37.5 35.0

105 kg 42.5 42.5 40.0 40.0 40.0 40.0 40.0 40.0 40.0 40.0 37.5 37.5 37.5 37.5 37.5

110 kg 42.5 42.5 42.5 42.5 42.5 42.5 40.0 40.0 40.0 40.0 40.0 40.0 40.0 37.5 37.5

115 kg 45.0 42.5 42.5 42.5 42.5 42.5 42.5 42.5 42.5 40.0 40.0 40.0 40.0 40.0 40.0

120 kg 45.0 45.0 45.0 45.0 45.0 42.5 42.5 42.5 42.5 42.5 42.5 42.5 40.0 40.0 40.0

125 kg 45.0 45.0 45.0 45.0 45.0 45.0 45.0 45.0 42.5 42.5 42.5 42.5 42.5 42.5 42.5

130 kg 47.5 47.5 47.5 47.5 45.0 45.0 45.0 45.0 45.0 45.0 45.0 42.5 42.5 42.5 42.5

135 kg 47.5 47.5 47.5 47.5 47.5 47.5 47.5 45.0 45.0 45.0 45.0 45.0 45.0 45.0 45.0

140 kg 50.0 50.0 50.0 47.5 47.5 47.5 47.5 47.5 47.5 47.5 45.0 45.0 45.0 45.0 45.0

145 kg 50.0 50.0 50.0 50.0 50.0 50.0 47.5 47.5 47.5 47.5 47.5 47.5 47.5 47.5 45.0

150 kg 52.5 52.5 50.0 50.0 50.0 50.0 50.0 50.0 50.0 47.5 47.5 47.5 47.5 47.5 47.5

HIV_positive

20yrs 25yrs 30yrs 35yrs 40yrs 45yrs 50yrs 55yrs 60yrs 65yrs 70yrs 75yrs 80yrs 85yrs 90yrs

35 kg 35.0 35.0 35.0 35.0 35.0 35.0 32.5 32.5 32.5 32.5 32.5 32.5 32.5 30.0 30.0

40 kg 37.5 35.0 35.0 35.0 35.0 35.0 35.0 35.0 35.0 32.5 32.5 32.5 32.5 32.5 32.5

45 kg 37.5 37.5 37.5 37.5 37.5 35.0 35.0 35.0 35.0 35.0 35.0 35.0 32.5 32.5 32.5

50 kg 37.5 37.5 37.5 37.5 37.5 37.5 37.5 37.5 35.0 35.0 35.0 35.0 35.0 35.0 35.0

55 kg 40.0 40.0 40.0 40.0 37.5 37.5 37.5 37.5 37.5 37.5 37.5 35.0 35.0 35.0 35.0

60 kg 40.0 40.0 40.0 40.0 40.0 40.0 40.0 37.5 37.5 37.5 37.5 37.5 37.5 37.5 35.0

65 kg 42.5 42.5 42.5 40.0 40.0 40.0 40.0 40.0 40.0 40.0 37.5 37.5 37.5 37.5 37.5

70 kg 42.5 42.5 42.5 42.5 42.5 42.5 40.0 40.0 40.0 40.0 40.0 40.0 40.0 40.0 37.5

75 kg 45.0 45.0 42.5 42.5 42.5 42.5 42.5 42.5 42.5 40.0 40.0 40.0 40.0 40.0 40.0

80 kg 45.0 45.0 45.0 45.0 45.0 42.5 42.5 42.5 42.5 42.5 42.5 42.5 42.5 40.0 40.0

85 kg 47.5 45.0 45.0 45.0 45.0 45.0 45.0 45.0 42.5 42.5 42.5 42.5 42.5 42.5 42.5

90 kg 47.5 47.5 47.5 47.5 45.0 45.0 45.0 45.0 45.0 45.0 45.0 45.0 42.5 42.5 42.5

95 kg 47.5 47.5 47.5 47.5 47.5 47.5 47.5 45.0 45.0 45.0 45.0 45.0 45.0 45.0 45.0

100 kg 50.0 50.0 50.0 47.5 47.5 47.5 47.5 47.5 47.5 47.5 47.5 45.0 45.0 45.0 45.0

105 kg 50.0 50.0 50.0 50.0 50.0 50.0 47.5 47.5 47.5 47.5 47.5 47.5 47.5 47.5 45.0

110 kg 52.5 52.5 50.0 50.0 50.0 50.0 50.0 50.0 50.0 50.0 47.5 47.5 47.5 47.5 47.5

115 kg 52.5 52.5 52.5 52.5 52.5 50.0 50.0 50.0 50.0 50.0 50.0 50.0 50.0 47.5 47.5

120 kg 55.0 52.5 52.5 52.5 52.5 52.5 52.5 52.5 52.5 50.0 50.0 50.0 50.0 50.0 50.0

125 kg 55.0 55.0 55.0 55.0 52.5 52.5 52.5 52.5 52.5 52.5 52.5 52.5 50.0 50.0 50.0

130 kg 55.0 55.0 55.0 55.0 55.0 55.0 55.0 55.0 52.5 52.5 52.5 52.5 52.5 52.5 52.5

135 kg 57.5 57.5 57.5 55.0 55.0 55.0 55.0 55.0 55.0 55.0 55.0 52.5 52.5 52.5 52.5

140 kg 57.5 57.5 57.5 57.5 57.5 57.5 57.5 55.0 55.0 55.0 55.0 55.0 55.0 55.0 52.5

145 kg 60.0 60.0 57.5 57.5 57.5 57.5 57.5 57.5 57.5 57.5 55.0 55.0 55.0 55.0 55.0

150 kg 60.0 60.0 60.0 60.0 60.0 60.0 57.5 57.5 57.5 57.5 57.5 57.5 57.5 55.0 55.0

HIV_unknown

20yrs 25yrs 30yrs 35yrs 40yrs 45yrs 50yrs 55yrs 60yrs 65yrs 70yrs 75yrs 80yrs 85yrs 90yrs

35 kg 30.0 30.0 30.0 30.0 27.5 27.5 27.5 27.5 27.5 27.5 27.5 27.5 25.0 25.0 25.0

40 kg 30.0 30.0 30.0 30.0 30.0 30.0 30.0 30.0 27.5 27.5 27.5 27.5 27.5 27.5 27.5

45 kg 32.5 32.5 32.5 30.0 30.0 30.0 30.0 30.0 30.0 30.0 30.0 27.5 27.5 27.5 27.5

50 kg 32.5 32.5 32.5 32.5 32.5 32.5 32.5 30.0 30.0 30.0 30.0 30.0 30.0 30.0 27.5

55 kg 35.0 35.0 32.5 32.5 32.5 32.5 32.5 32.5 32.5 32.5 30.0 30.0 30.0 30.0 30.0

60 kg 35.0 35.0 35.0 35.0 35.0 35.0 32.5 32.5 32.5 32.5 32.5 32.5 32.5 30.0 30.0

65 kg 37.5 35.0 35.0 35.0 35.0 35.0 35.0 35.0 35.0 32.5 32.5 32.5 32.5 32.5 32.5

70 kg 37.5 37.5 37.5 37.5 37.5 35.0 35.0 35.0 35.0 35.0 35.0 35.0 32.5 32.5 32.5

75 kg 37.5 37.5 37.5 37.5 37.5 37.5 37.5 37.5 35.0 35.0 35.0 35.0 35.0 35.0 35.0

80 kg 40.0 40.0 40.0 40.0 37.5 37.5 37.5 37.5 37.5 37.5 37.5 35.0 35.0 35.0 35.0

85 kg 40.0 40.0 40.0 40.0 40.0 40.0 40.0 37.5 37.5 37.5 37.5 37.5 37.5 37.5 37.5

90 kg 42.5 42.5 42.5 40.0 40.0 40.0 40.0 40.0 40.0 40.0 37.5 37.5 37.5 37.5 37.5

95 kg 42.5 42.5 42.5 42.5 42.5 42.5 40.0 40.0 40.0 40.0 40.0 40.0 40.0 40.0 37.5

100 kg 45.0 45.0 42.5 42.5 42.5 42.5 42.5 42.5 42.5 40.0 40.0 40.0 40.0 40.0 40.0

105 kg 45.0 45.0 45.0 45.0 45.0 42.5 42.5 42.5 42.5 42.5 42.5 42.5 42.5 40.0 40.0

110 kg 47.5 45.0 45.0 45.0 45.0 45.0 45.0 45.0 42.5 42.5 42.5 42.5 42.5 42.5 42.5

115 kg 47.5 47.5 47.5 47.5 45.0 45.0 45.0 45.0 45.0 45.0 45.0 45.0 42.5 42.5 42.5

120 kg 47.5 47.5 47.5 47.5 47.5 47.5 47.5 45.0 45.0 45.0 45.0 45.0 45.0 45.0 45.0

125 kg 50.0 50.0 50.0 47.5 47.5 47.5 47.5 47.5 47.5 47.5 47.5 45.0 45.0 45.0 45.0

130 kg 50.0 50.0 50.0 50.0 50.0 50.0 47.5 47.5 47.5 47.5 47.5 47.5 47.5 47.5 45.0

135 kg 52.5 52.5 50.0 50.0 50.0 50.0 50.0 50.0 50.0 50.0 47.5 47.5 47.5 47.5 47.5

140 kg 52.5 52.5 52.5 52.5 52.5 50.0 50.0 50.0 50.0 50.0 50.0 50.0 50.0 47.5 47.5

145 kg 55.0 52.5 52.5 52.5 52.5 52.5 52.5 52.5 52.5 50.0 50.0 50.0 50.0 50.0 50.0

150 kg 55.0 55.0 55.0 55.0 52.5 52.5 52.5 52.5 52.5 52.5 52.5 52.5 50.0 50.0 50.0

#### Target INR range 2.5 to 3.5

HIV_negative

20yrs 25yrs 30yrs 35yrs 40yrs 45yrs 50yrs 55yrs 60yrs 65yrs 70yrs 75yrs 80yrs 85yrs 90yrs

35 kg 35.0 32.5 32.5 32.5 32.5 32.5 32.5 32.5 32.5 30.0 30.0 30.0 30.0 30.0 30.0

40 kg 35.0 35.0 35.0 35.0 32.5 32.5 32.5 32.5 32.5 32.5 32.5 32.5 30.0 30.0 30.0

45 kg 35.0 35.0 35.0 35.0 35.0 35.0 35.0 35.0 32.5 32.5 32.5 32.5 32.5 32.5 32.5

50 kg 37.5 37.5 37.5 35.0 35.0 35.0 35.0 35.0 35.0 35.0 35.0 32.5 32.5 32.5 32.5

55 kg 37.5 37.5 37.5 37.5 37.5 37.5 37.5 35.0 35.0 35.0 35.0 35.0 35.0 35.0 32.5

60 kg 40.0 40.0 37.5 37.5 37.5 37.5 37.5 37.5 37.5 37.5 35.0 35.0 35.0 35.0 35.0

65 kg 40.0 40.0 40.0 40.0 40.0 40.0 37.5 37.5 37.5 37.5 37.5 37.5 37.5 35.0 35.0

70 kg 42.5 40.0 40.0 40.0 40.0 40.0 40.0 40.0 40.0 37.5 37.5 37.5 37.5 37.5 37.5

75 kg 42.5 42.5 42.5 42.5 42.5 40.0 40.0 40.0 40.0 40.0 40.0 40.0 37.5 37.5 37.5

80 kg 42.5 42.5 42.5 42.5 42.5 42.5 42.5 42.5 40.0 40.0 40.0 40.0 40.0 40.0 40.0

85 kg 45.0 45.0 45.0 45.0 42.5 42.5 42.5 42.5 42.5 42.5 42.5 40.0 40.0 40.0 40.0

90 kg 45.0 45.0 45.0 45.0 45.0 45.0 45.0 42.5 42.5 42.5 42.5 42.5 42.5 42.5 42.5

95 kg 47.5 47.5 47.5 45.0 45.0 45.0 45.0 45.0 45.0 45.0 42.5 42.5 42.5 42.5 42.5

100 kg 47.5 47.5 47.5 47.5 47.5 47.5 45.0 45.0 45.0 45.0 45.0 45.0 45.0 45.0 42.5

105 kg 50.0 50.0 47.5 47.5 47.5 47.5 47.5 47.5 47.5 45.0 45.0 45.0 45.0 45.0 45.0

110 kg 50.0 50.0 50.0 50.0 50.0 47.5 47.5 47.5 47.5 47.5 47.5 47.5 47.5 45.0 45.0

115 kg 52.5 50.0 50.0 50.0 50.0 50.0 50.0 50.0 47.5 47.5 47.5 47.5 47.5 47.5 47.5

120 kg 52.5 52.5 52.5 52.5 50.0 50.0 50.0 50.0 50.0 50.0 50.0 50.0 47.5 47.5 47.5

125 kg 52.5 52.5 52.5 52.5 52.5 52.5 52.5 50.0 50.0 50.0 50.0 50.0 50.0 50.0 50.0

130 kg 55.0 55.0 55.0 52.5 52.5 52.5 52.5 52.5 52.5 52.5 52.5 50.0 50.0 50.0 50.0

135 kg 55.0 55.0 55.0 55.0 55.0 55.0 52.5 52.5 52.5 52.5 52.5 52.5 52.5 52.5 50.0

140 kg 57.5 57.5 55.0 55.0 55.0 55.0 55.0 55.0 55.0 55.0 52.5 52.5 52.5 52.5 52.5

145 kg 57.5 57.5 57.5 57.5 57.5 55.0 55.0 55.0 55.0 55.0 55.0 55.0 55.0 52.5 52.5

150 kg 60.0 57.5 57.5 57.5 57.5 57.5 57.5 57.5 57.5 55.0 55.0 55.0 55.0 55.0 55.0

HIV_positive

20yrs 25yrs 30yrs 35yrs 40yrs 45yrs 50yrs 55yrs 60yrs 65yrs 70yrs 75yrs 80yrs 85yrs 90yrs

35 kg 42.5 42.5 42.5 42.5 42.5 40.0 40.0 40.0 40.0 40.0 40.0 40.0 40.0 37.5 37.5

40 kg 45.0 42.5 42.5 42.5 42.5 42.5 42.5 42.5 40.0 40.0 40.0 40.0 40.0 40.0 40.0

45 kg 45.0 45.0 45.0 45.0 42.5 42.5 42.5 42.5 42.5 42.5 42.5 42.5 40.0 40.0 40.0

50 kg 45.0 45.0 45.0 45.0 45.0 45.0 45.0 42.5 42.5 42.5 42.5 42.5 42.5 42.5 42.5

55 kg 47.5 47.5 47.5 45.0 45.0 45.0 45.0 45.0 45.0 45.0 45.0 42.5 42.5 42.5 42.5

60 kg 47.5 47.5 47.5 47.5 47.5 47.5 45.0 45.0 45.0 45.0 45.0 45.0 45.0 45.0 42.5

65 kg 50.0 50.0 47.5 47.5 47.5 47.5 47.5 47.5 47.5 47.5 45.0 45.0 45.0 45.0 45.0

70 kg 50.0 50.0 50.0 50.0 50.0 47.5 47.5 47.5 47.5 47.5 47.5 47.5 47.5 45.0 45.0

75 kg 52.5 50.0 50.0 50.0 50.0 50.0 50.0 50.0 50.0 47.5 47.5 47.5 47.5 47.5 47.5

80 kg 52.5 52.5 52.5 52.5 50.0 50.0 50.0 50.0 50.0 50.0 50.0 50.0 47.5 47.5 47.5

85 kg 52.5 52.5 52.5 52.5 52.5 52.5 52.5 52.5 50.0 50.0 50.0 50.0 50.0 50.0 50.0

90 kg 55.0 55.0 55.0 52.5 52.5 52.5 52.5 52.5 52.5 52.5 52.5 50.0 50.0 50.0 50.0

95 kg 55.0 55.0 55.0 55.0 55.0 55.0 55.0 52.5 52.5 52.5 52.5 52.5 52.5 52.5 50.0

100 kg 57.5 57.5 55.0 55.0 55.0 55.0 55.0 55.0 55.0 55.0 52.5 52.5 52.5 52.5 52.5

105 kg 57.5 57.5 57.5 57.5 57.5 57.5 55.0 55.0 55.0 55.0 55.0 55.0 55.0 52.5 52.5

110 kg 60.0 57.5 57.5 57.5 57.5 57.5 57.5 57.5 57.5 55.0 55.0 55.0 55.0 55.0 55.0

115 kg 60.0 60.0 60.0 60.0 60.0 57.5 57.5 57.5 57.5 57.5 57.5 57.5 55.0 55.0 55.0

120 kg 60.0 60.0 60.0 60.0 60.0 60.0 60.0 60.0 57.5 57.5 57.5 57.5 57.5 57.5 57.5

125 kg 62.5 62.5 62.5 62.5 60.0 60.0 60.0 60.0 60.0 60.0 60.0 57.5 57.5 57.5 57.5

130 kg 62.5 62.5 62.5 62.5 62.5 62.5 62.5 60.0 60.0 60.0 60.0 60.0 60.0 60.0 60.0

135 kg 65.0 65.0 65.0 62.5 62.5 62.5 62.5 62.5 62.5 62.5 60.0 60.0 60.0 60.0 60.0

140 kg 65.0 65.0 65.0 65.0 65.0 65.0 62.5 62.5 62.5 62.5 62.5 62.5 62.5 62.5 60.0

145 kg 67.5 67.5 65.0 65.0 65.0 65.0 65.0 65.0 65.0 62.5 62.5 62.5 62.5 62.5 62.5

150 kg 67.5 67.5 67.5 67.5 67.5 65.0 65.0 65.0 65.0 65.0 65.0 65.0 65.0 62.5 62.5

HIV_unknown

20yrs 25yrs 30yrs 35yrs 40yrs 45yrs 50yrs 55yrs 60yrs 65yrs 70yrs 75yrs 80yrs 85yrs 90yrs

35 kg 37.5 37.5 37.5 37.5 35.0 35.0 35.0 35.0 35.0 35.0 35.0 32.5 32.5 32.5 32.5

40 kg 37.5 37.5 37.5 37.5 37.5 37.5 37.5 35.0 35.0 35.0 35.0 35.0 35.0 35.0 35.0

45 kg 40.0 40.0 40.0 37.5 37.5 37.5 37.5 37.5 37.5 37.5 35.0 35.0 35.0 35.0 35.0

50 kg 40.0 40.0 40.0 40.0 40.0 40.0 37.5 37.5 37.5 37.5 37.5 37.5 37.5 37.5 35.0

55 kg 42.5 42.5 40.0 40.0 40.0 40.0 40.0 40.0 40.0 37.5 37.5 37.5 37.5 37.5 37.5

60 kg 42.5 42.5 42.5 42.5 42.5 40.0 40.0 40.0 40.0 40.0 40.0 40.0 40.0 37.5 37.5

65 kg 45.0 42.5 42.5 42.5 42.5 42.5 42.5 42.5 40.0 40.0 40.0 40.0 40.0 40.0 40.0

70 kg 45.0 45.0 45.0 45.0 42.5 42.5 42.5 42.5 42.5 42.5 42.5 42.5 40.0 40.0 40.0

75 kg 45.0 45.0 45.0 45.0 45.0 45.0 45.0 42.5 42.5 42.5 42.5 42.5 42.5 42.5 42.5

80 kg 47.5 47.5 47.5 45.0 45.0 45.0 45.0 45.0 45.0 45.0 45.0 42.5 42.5 42.5 42.5

85 kg 47.5 47.5 47.5 47.5 47.5 47.5 45.0 45.0 45.0 45.0 45.0 45.0 45.0 45.0 42.5

90 kg 50.0 50.0 47.5 47.5 47.5 47.5 47.5 47.5 47.5 47.5 45.0 45.0 45.0 45.0 45.0

95 kg 50.0 50.0 50.0 50.0 50.0 47.5 47.5 47.5 47.5 47.5 47.5 47.5 47.5 45.0 45.0

100 kg 52.5 50.0 50.0 50.0 50.0 50.0 50.0 50.0 50.0 47.5 47.5 47.5 47.5 47.5 47.5

105 kg 52.5 52.5 52.5 52.5 52.5 50.0 50.0 50.0 50.0 50.0 50.0 50.0 47.5 47.5 47.5

110 kg 52.5 52.5 52.5 52.5 52.5 52.5 52.5 52.5 50.0 50.0 50.0 50.0 50.0 50.0 50.0

115 kg 55.0 55.0 55.0 55.0 52.5 52.5 52.5 52.5 52.5 52.5 52.5 50.0 50.0 50.0 50.0

120 kg 55.0 55.0 55.0 55.0 55.0 55.0 55.0 52.5 52.5 52.5 52.5 52.5 52.5 52.5 50.0

125 kg 57.5 57.5 57.5 55.0 55.0 55.0 55.0 55.0 55.0 55.0 52.5 52.5 52.5 52.5 52.5

130 kg 57.5 57.5 57.5 57.5 57.5 57.5 55.0 55.0 55.0 55.0 55.0 55.0 55.0 52.5 52.5

135 kg 60.0 60.0 57.5 57.5 57.5 57.5 57.5 57.5 57.5 55.0 55.0 55.0 55.0 55.0 55.0

140 kg 60.0 60.0 60.0 60.0 60.0 57.5 57.5 57.5 57.5 57.5 57.5 57.5 57.5 55.0 55.0

145 kg 62.5 60.0 60.0 60.0 60.0 60.0 60.0 60.0 57.5 57.5 57.5 57.5 57.5 57.5 57.5

150 kg 62.5 62.5 62.5 62.5 60.0 60.0 60.0 60.0 60.0 60.0 60.0 60.0 57.5 57.5 57.5

### Example patients

| **Clinical variables** | **Weekly dose (using equation)** | **3-day dose** | **Schedule A** | **Schedule B** |
| --- | --- | --- | --- | --- |
| Age = 36 years, Gender = male, Weight = 62 kg, INR Target range = 2–3, HIV negative | = 20.2832 – 0.0656 x 36 + 0.2178 x 62  = 31.4 mg  *[Becomes 32.5 mg when rounded to the nearest 2.5 mg]* | = 32.5 * 3 /7  = 13.9  *[Becomes 15 mg when rounded up to the nearest 2.5 mg]* | 5 + 5 + 5 | 5 + 5 + 5 |
| Age = 39 years, Gender = female, Weight = 69 kg, INR Target range = 2.5–3.5, HIV positive | = 20.2832 – 0.0656 x 39 + 0.2178 x 69 + 7.3190 + 8.7973  = 48.9 mg  *[Becomes 50 mg when rounded to the nearest 2.5 mg]* | = 50 * 3 /7  = 21.4  *[Becomes* 22.5 *mg when rounded up to the nearest 2.5 mg]* | 7.5 + 7.5 + 7.5 | 5 + 10 + 7.5 |
| Age = 30 years, Gender = female, Weight = 116 kg, INR Target range = 2.5–3.5, HIV negative | = 20.2832 – 0.0656 x 30 + 0.2178 x 116 + 7.3190  = 50.9 mg  *[Becomes 50 mg when rounded to the nearest 2.5 mg]* | = 50 * 3 /7  = 22.5 mg | 7.5 + 7.5 + 7.5 | 5 + 10 + 7.5 |
