# Supplementary material for: A “Bundle of Care” to Improve Anticoagulation Control in Patients Receiving Warfarin in Uganda and South Africa: Protocol for an Implementation Study
Source: JMIR Res Protoc. 2023 Jul 19;12:e46710. doi: 10.2196/46710 (PMC10398551; doi:10.2196/46710)
Supplement: Multimedia Appendix 2 [file resprot_v12i1e46710_app2.docx]

## Appendix 2. Warfarin Dosing Algorithms for other ethnicities

In a recent systematic review of >378 warfarin dosing algorithms (17), the two most externally validated clinical algorithms were the Gage (2008) and International Warfarin Pharmacogenetics Consortium (IWPC, 2009) equations, validated 14 and 13 times, respectively. The IWPC (2009) dose-initiation algorithm was preferred to Gage (2008) because it additionally incorporates ‘Asian’ and ‘Missing or Mixed race’ categories unlike the Gage (2008) algorithm that only caters for the ‘White’ and ‘Black’ racial categories. Excluding the patients whose self-reported race is Black African (for which we have designed a separate clinical algorithm), the IWPC dose-initiation equation becomes:

Square root of weekly warfarin dose = 4.0376 – 0.2546 (age in decades) + 0.0118 (height in cm) + 0.0134 (weight in kg) – 0.6752 (1 if self-reported race is Asian, otherwise zero) + 0.0443 (1 if self-reported race is unspecified or mixed, otherwise zero) + 1.2799 (1 if patient taking carbamazepine, phenytoin, rifampin, or rifampicin, otherwise zero) – 0.5695 (1 if patient taking amiodarone, otherwise zero)

The output of the above algorithm must be squared to compute the weekly dose in mg. After obtaining the weekly dose, the 3-day dosing schedules will be calculated as reported for the Black African algorithm.
